# Trends in food insecurity for adults with cardiometabolic disease in the United States: 2005-2012

**DOI:** 10.1371/journal.pone.0179172

**Published:** 2017-06-07

**Authors:** Seth A. Berkowitz, Theodore S. Z. Berkowitz, James B. Meigs, Deborah J. Wexler

**Affiliations:** 1 Division of General Internal Medicine, Massachusetts General Hospital, Boston, Massachusetts, United States of America; 2 Diabetes Unit, Massachusetts General Hospital, Boston, Massachusetts, United States of America; 3 Harvard Medical School, Boston, Massachusetts, United States of America; 4 Center for Health Services Research in Primary Care, Durham Veterans Affairs Medical Center, Durham, North Carolina, United States of America; Nagoya University, JAPAN

## Abstract

**Background:**

Food insecurity, the uncertain ability to access adequate food, can limit adherence to dietary measures needed to prevent and manage cardiometabolic conditions. However, little is known about temporal trends in food insecurity among those with diet-sensitive cardiometabolic conditions.

**Methods:**

We used data from the Continuous National Health and Nutrition Examination Survey (NHANES) 2005–2012, analyzed in 2015–2016, to calculate trends in age-standardized rates of food insecurity for those with and without the following diet-sensitive cardiometabolic conditions: diabetes mellitus, hypertension, coronary heart disease, congestive heart failure, and obesity.

**Results:**

21,196 NHANES participants were included from 4 waves (4,408 in 2005–2006, 5,607 in 2007–2008, 5,934 in 2009–2010, and 5,247 in 2011–2012). 56.2% had at least one cardiometabolic condition, 24.4% had 2 or more, and 8.5% had 3 or more. The overall age-standardized rate of food insecurity doubled during the study period, from 9.06% in 2005–2006 to 10.82% in 2007–2008 to 15.22% in 2009–2010 to 18.33% in 2011–2012 (p for trend < .001). The average annual percentage change in food insecurity for those with a cardiometabolic condition during the study period was 13.0% (95% CI 7.5% to 18.6%), compared with 5.8% (95% CI 1.8% to 10.0%) for those without a cardiometabolic condition, (parallelism test p = .13). Comparing those with and without the condition, age-standardized rates of food insecurity were greater in participants with diabetes (19.5% vs. 11.5%, p < .0001), hypertension (14.1% vs. 11.1%, p = .0003), coronary heart disease (20.5% vs. 11.9%, p < .001), congestive heart failure (18.4% vs. 12.1%, p = .004), and obesity (14.3% vs. 11.1%, p < .001).

**Conclusions:**

Food insecurity doubled to historic highs from 2005–2012, particularly affecting those with diet-sensitive cardiometabolic conditions. Since adherence to specific dietary recommendations is a foundation of the prevention and treatment of cardiometabolic disease, these results have important implications for clinical management and public health.

## Introduction

Cardiometabolic diseases, including diabetes, coronary heart disease, and congestive heart failure, and cardiovascular risk factors, such as hypertension and obesity, are leading causes of morbidity and mortality in the United States.[1] One common thread among these conditions is diet-sensitivity—the cornerstone of their prevention and clinical management is dietary modification, such increased consumption of dietary fiber, whole grains, lean proteins, and unsaturated fats, along with salt reduction.[2–6]

While following a healthy diet can be difficult for anyone, those with food insecurity face particular challenges.[7] Food insecurity is defined as uncertain ability to obtain nutritious foods in socially acceptable ways.[8]. Food insecurity is associated with poor diet quality, in part related to the greater expense of foods such as fresh produce and whole grains, compared with shelf-stable foods high in refined carbohydrates and sodium.[7, 9] Previous research has shown that food insecurity is associated with several cardiometabolic conditions, including diabetes and obesity.[10–14] Food insecurity likely has a bi-directional relationship with cardiometabolic disease: it may increase risk for and poor control of cardiometabolic disease through poor diet, and cardiometabolic disease may increase food insecurity through diminished ability to work and competing medical expenses.[7, 15]

The chief method for measuring food insecurity in American households is through the Current Population Survey.[16] From 1995, the inception of widespread measurement, until 2007 the prevalence of food insecurity was relatively constant, hovering around 11%, and peaking at 12% in 1996.[16] A limitation of the Current Population Survey data, however, is that it does not collect detailed clinical information. For this reason, it does not provide sufficient information about trends in food insecurity among those with diet-sensitive cardiometabolic conditions. This is important since adults with cardiometabolic conditions are at highest risk for poor health related to food insecurity. Understanding trends in food insecurity among those with specific cardiometabolic conditions is vital for both clinical management and public health. Therefore, we sought to determine trends in food insecurity and use of programs to address it among those with cardiometabolic disease in the U.S. from 2005–2012. Owing to the economic recession that began in late 2007[17], we hypothesized that food insecurity would rise significantly during the study period. Because cardiometabolic conditions often interfere with the ability to work, and bring attendant medical expenses, both of which can further exacerbate food insecurity[7], we hypothesized that those with cardiometabolic conditions would experience even greater increases in food insecurity than those without these conditions.

## Methods

The Human Research Committee at Partners Healthcare exempted this study from institutional review board approval.

### Data source

To address these major questions in the epidemiology of food insecurity, we used data from the Continuous National Health and Nutrition Examination Survey (NHANES), covering the years 2005–2012. These years were chosen because of consistent collection of relevant exposure and outcome data over the time period, with 2011–2012 being the most recent time period for which data were available at the time of our analysis in 2015–2016. NHANES is a nationally representative, repeated cross-section, multi-stage probability sample of the non-institutionalized population of the United States.[18] NHANES respondents complete a home interview followed by a laboratory and anthropometric examination in a mobile examination center. All non-pregnant adult participants (age > 20 years) who completed the examination were included in the study. Detailed data collection methods and documentation are available through the NHANES website (http://www.cdc.gov/nchs/nhanes/nhanes_questionnaires.htm).

This study made use of third-party data from the United States’ National Center for Health Statistics, which can be freely downloaded under a data use agreement at: https://wwwn.cdc.gov/nchs/nhanes/Default.aspx.

### Cardiometabolic conditions

We studied the following cardiometabolic conditions that have been associated with food insecurity and that are sensitive to diet with regard to developing the condition, and/or require adherence to specific dietary recommendations as a key part of treatment (S1 Table for specific case definitions): diabetes mellitus, hypertension, coronary heart disease, congestive heart failure, and obesity.[2–7, 10, 11, 19, 20] For example, congestive heart failure was included because exacerbations including hospital admissions are highly sensitive to dietary composition, and following a low-sodium diet is a cornerstone of management. Cheaper, processed foods that may be eaten preferentially by those with food insecurity are often particularly high in sodium. Further, other conditions associated with food insecurity, such as hypertension and coronary heart disease, may lead to congestive heart failure. Other conditions similarly include specific dietary recommendations as part of management and deteriorate in the setting of diet non-adherence. As in prior analyses of NHANES data, the presence of cardiometabolic conditions was indicated by affirmative response to previously validated self-report items, laboratory values, physical examination findings, and/or medication use.[19, 21–23] In addition, we examined three subgroups at high risk for complications among those with particular conditions: uncontrolled hemoglobin A1c (HbA1c) among those with diabetes (HbA1c > 9.0%), uncontrolled low-density lipoprotein (LDL) cholesterol among those with diabetes or coronary heart disease (LDL > 100 mg/dL), and uncontrolled hypertension among those with hypertension (defined as systolic blood pressure > 140 mm Hg or diastolic blood pressure > 90 mm Hg), using NHANES laboratory or examination data.

### Food insecurity

Food insecurity was defined using the 10 adult referenced items of the United States Department of Agriculture’s (USDA’s) Food Security Survey module within NHANES.[19, 24] An example item is: “I worried whether my food would run out before I got money to buy more”.[24] The same items were used throughout the study period. Using standard scoring, three or more affirmative responses indicated food insecurity, while fewer than three affirmative responses indicated food security.[24] Owing to sample size issues, we did not further characterize food insecurity as low or very low food security.

### The hunger safety net

Given the known associations between food insecurity and health, interest is growing in ‘linkage’ interventions—programs that link patients to nutrition assistance via the hunger safety net in order to aid chronic disease management.[25] In order to understand the potential for linkage interventions, we sought to examine participation in two key components of the hunger safety net: the Supplemental Nutrition Assistance Program (SNAP, formerly the Food Stamp Program) and use of emergency food sources such as food pantries and soup kitchens.

### SNAP

SNAP is the largest anti-hunger program in the United States.[26] Income is the primary eligibility criterion. Similar to prior studies[27–29], we considered a participant income-eligible for SNAP if their income, adjusted for household size, was 130% or less of the federal poverty level in the year of NHANES participation. It is important to note that factors in addition to income level can be used to determine SNAP eligibility, that not all of these factors are captured in NHANES, and that criteria can vary over time and across U.S. states.[26] Further, participant state of residence is masked to protect privacy. For these reasons, we could only determine if a participant was income-eligible for SNAP. SNAP use was determined by self-report.

### Emergency food use

In addition to government programs to combat food insecurity, the hunger safety net includes organizations that provide food as charity, such as food banks and congregate meal sites. To assess use of these emergency sources of food, NHANES participants were asked whether they obtained food “from a church, food pantry, food bank, or soup kitchen” in the preceding 12 months.

### Statistical analysis

The goal of this project was to help determine food insecurity trends, whether those trends are similar in those with and without cardiometabolic conditions of interest, whether trends improved after the economic recession ended in 2009[17], and to estimate use of the hunger safety net. To do this, we conducted several analyses using standard approaches. First, our primary analyses present age-standardized rates of food insecurity, by clinical condition, in four NHANES periods: 2005–2006, 2007–2008, 2009–2010, and 2011–2012 and test whether these rates are different for those with, versus without, the clinical condition, using chi-squared tests. Results are age-standardized (to the Census 2000 population following National Center for Health Statistics guidance[30]) as most cardiometabolic conditions are highly age-related. Next, we examined whether these rates increased over time, using a permutation test for trend testing in Joinpoint Trend Analysis Software (Version 4.2.0.2 http://surveillance.cancer.gov/joinpoint/).[31] Next, to examine hunger safety net use, we analyzed rates of SNAP and emergency food use, trends in SNAP and emergency food use, and whether these rates differed between those with and without a particular cardiometabolic condition. Finally, because food insecurity is known to disproportionately affect particular demographic groups[8], we calculated rates of food insecurity by cardiometabolic condition, stratified by gender, race/ethnicity, and educational attainment. These are reported in the Supplementary Appendix (S2 Table).

All analyses used appropriate survey design information and sampling weights to account for the complex sampling strategy.[18] To test statistical significance, we used chi-squared tests for categorical variables, linear regression for continuous variables, A p-value of <0.05 was taken to indicate statistical significance. All analyses, other than the trend testing described above, were conducted in SAS version 9.4 (SAS Institute, Cary, NC) and figures were created with the ggplot2 package, version 2.1.0[32] using R software, version 3.2.3.[33]

## Results

Overall, 21,196 NHANES participants were included in the study, 4,408 in the 2005–2006 wave, 5,607 in 2007–2008, 5,934 in 2009–2010, and 5,247 in 2011–2012 (Table 1). Among these participants, 56.2% had at least one cardiometabolic condition, 24.4% had 2 or more, and 8.5% had 3 or more.

**Table 1:** Demographic characteristics of NHANES participants.

	All Participants (2005–2012)		2005–2006		2007–2008		2009–2010		2011–2012	
	Food Secure	Food Insecure	Food Secure	Food Insecure	Food Secure	Food Insecure	Food Secure	Food Insecure	Food Secure	Food Insecure
	N = 17657	N = 3539	N = 3857	N = 551	N = 4801	N = 806	N = 4791	N = 1143	N = 4208	N = 1039
Age, y (SE)	48.0 (0.34)	41.5 (0.48)	47.6 (0.80)	40.6 (0.85)	47.6 (0.42)	41.6 (0.92)	48.1 (0.54)	41.1 (0.80)	48.5 (0.85)	42.2 (1.04)
Female, %	51.2	51.6	51.0	50.3	51.3	52.3	51.1	52.7	51.6	51.1
Race/Ethnicity, %										
Non-Hispanic White	72.3	46.6	74.9	45.9	72.0	50.7	72.3	40.9	69.8	48.9
Non-Hispanic Black	10.2	19.8	10.5	21.8	10.6	16.9	9.6	23.2	10.2	18.0
Hispanic	11.0	26.5	9.6	22.5	11.5	26.2	10.9	29.8	12.1	26.2
Asian/Multi-/Other	6.5	7.1	4.9	9.9	5.9	6.2	7.1	6.0	7.9	7.0
< High School Diploma, %	15.90	37.1	15.8	35.4	18.2	41.3	15.6	41.1	13.9	32.2
Ratio of Income to FPL (SE)	3.21 (0.04)	1.42 (0.04)	3.27 (0.06)	1.48 (0.06)	3.19 (0.09)	1.49 (0.09)	3.23 (0.05)	1.32 (0.09)	3.15 (0.09)	1.42 (0.07)
Cardiometabolic Conditions, %										
Diabetes Mellitus	11.3	14.3	10.0	12.2	11.9	13.9	11.7	13.9	11.6	16.2
Hypertension	38.1	34.3	38.6	30.2	38.2	35.2	37.4	32.8	38.3	37.0
Coronary Heart Disease	5.6	6.5	6.2	6.4	5.2	8.5	5.7	5.1	5.3	6.4
Congestive Heart Failure	2.4	2.8	2.5	3.4	2.3	3.2	2.1	1.4	2.7	3.5
Obesity	33.8	39.9	34.1	34.4	33.1	38.3	35.6	40.7	33.0	43.1
≥ 1 condition	56.1	57.3	57.1	50.0	56.0	56.3	55.8	56.1	55.4	62.7
≥ 2 conditions	24.1	26.4	24.2	23.5	23.9	26.1	24.6	25.5	23.8	28.8
≥ 3 conditions	8.2	10.0	7.6	7.8	8.0	11.6	8.9	9.3	8.4	10.8

FPL = Federal Poverty Level

### Food insecurity

Combining all time periods, and comparing those with and without the condition, age-standardized rates of food insecurity were greater in participants with diabetes (19.5% vs. 11.5%, p < .001), hypertension (14.1% vs. 11.1%, p < .001), coronary heart disease (20.5% vs. 11.9%, p < .001), congestive heart failure (18.4% vs. 12.1%, p = .004) and obesity (14.3% vs. 11.1%, p < .001). Overall, food insecurity was more common in non-Hispanic Black (20.9%) and Hispanic (24.6%) than non-Hispanic White (8.1%) participants (p< .001). Food insecurity was also more common in participants with less than high school diploma educational attainment (24.1% vs. 9.2%, p < .001) but was similar in men (11.9%) and women (12.1%) (p = .14).

Among participants with diabetes, food insecurity was more common in those with uncontrolled diabetes (HbA1c>9), compared with those with better glycemic control (HbA1c<9), (29.6% vs. 17.9%, p < .001). However, among participants with coronary heart disease or diabetes, there was no statistically significant difference in food insecurity prevalence in those with uncontrolled (LDL > 100 mg /dL), compared with controlled, LDL cholesterol (22.3% vs. 15.8%, p = .08). Similarly, among participants with hypertension, food insecurity was not significantly more common in those with uncontrolled (SBP > 140 mm Hg or DBP > 90 mm Hg), compared with controlled, hypertension (13.3% vs. 14.6%, p = .42).

**Fig 1:**
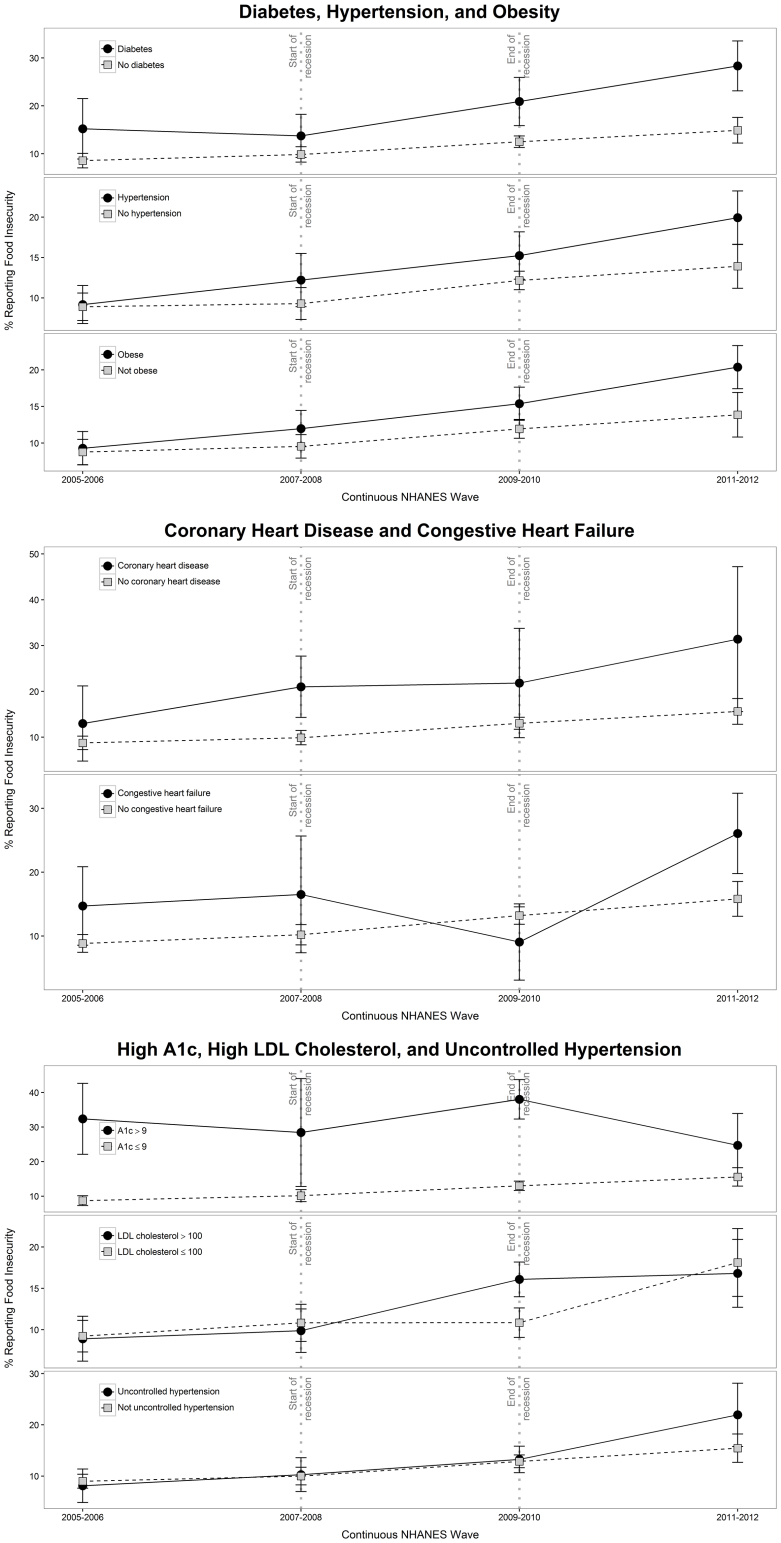
Trends in food insecurity prevalence among those with and without: Diabetes, hypertension, and obesity (top), coronary heart disease and congestive heart failure (middle) and uncontrolled hemoglobin A1c, low-density lipoprotein cholesterol, and hypertension (bottom).

Among all included participants, age-standardized rates of food insecurity increased during the study period, from 9.06% in 2005–2006 to 10.82% in 2007–2008 to 15.22% in 2009–2010 to 18.33% in 2011–2012 (p for trend < .001) (Fig 1). Age-standardized rates of food insecurity by condition and time period are presented in Table 2, and rates stratified by gender, race/ethnicity, and education are presented in S2 Table. The average annual percentage change (APC) in food insecurity for those with a cardiometabolic condition during the study period was 13.0% (95% Confidence Interval [CI] 7.5% to 18.6%), representing significant year-over-year increase in food insecurity from 2005 to 2012. For those without a cardiometabolic condition, the average APC was 5.8% (95% CI 1.8% to 10.0%) (S3 Table). There was no evidence of a significantly different trend for those with, versus without, a cardiometabolic condition, meaning that the increase in food insecurity occurred roughly in parallel (test for parallelism p-value = .13). Looking at the post-recession period, there was no evidence for a deflection in the trend of rising food insecurity.

**Table 2:** Food insecurity trends by NHANES wave.

	2005–2006			2007–2008			2009–2010			2011–2012		
	N	Age-standardized % Reporting Food Insecurity (SE)	p	N	Age-standardized % Reporting Food Insecurity (SE)	p	N	Age-standardized % Reporting Food Insecurity (SE)	p	N	Age-standardized % Reporting Food Insecurity (SE)	p
*Diabetes Mellitus*												
With	91	15.2 (3.2)	0.04	149	13.7 (2.3)	0.07	195	20.9 (2.6)	0.002	213	28.3 (2.7)	<0.001
Without	460	8.6 (0.8)	--	657	9.8 (0.8)	--	948	12.5 (0.6)	--	826	14.9 (1.4)	--
*Hypertension*												
With	204	9.2 (1.2)	0.97	340	12.2 (1.7)	0.11	431	15.2 (1.5)	0.04	443	19.9 (1.7)	<0.001
Without	347	8.9 (0.9)	--	466	9.3 (1.0)	--	712	12.2 (0.6)	--	596	13.9 (1.4)	--
*Coronary Heart Disease*												
With	51	13.0 (4.2)	0.34	78	21.0 (3.4)	0.001	79	21.8 (6.1)	0.14	71	31.4 (8.1)	0.06
Without	500	8.8 (0.7)	--	728	9.9 (0.8)	--	1064	13.0 (0.7)	--	968	15.6 (1.4)	--
*Congestive Heart Failure*												
With	23	14.7 (3.1)	0.09	34	16.5 (4.7)	0.07	25	9.1 (3.0)	0.31	45	26.1 (3.2)	<0.001
Without	528	8.8 (0.7)	--	772	10.2 (0.8)	--	1118	13.2 (0.7)	--	994	15.8 (1.4)	--
*Obesity*												
With	200	9.3 (1.2)	0.60	317	12.0 (1.3)	0.007	480	15.4 (1.2)	0.003	445	20.4 (1.5)	<0.001
Without	338	8.8 (0.9)	--	476	9.6 (0.8)	--	647	11.9 (0.7)	--	577	13.9 (1.6)	--
*HbA1c > 9%*^a^												
With	24	32.4 (5.2)	<0.001	30	28.4 (8.0)	0.02	40	38.0 (2.9)	<0.001	39	24.7 (4.7)	0.03
Without	63	8.7 (0.7)	--	106	10.1 (0.9)	--	136	13.0 (0.7)	--	160	15.6 (1.4)	--
*LDL > 100 mg/dL*^b^												
With	41	8.9 (1.4)	0.88	57	9.9 (1.3)	0.69	60	16.1 (1.1)	<0.001	60	16.8 (2.1)	0.74
Without	19	9.2 (1.0)	--	29	10.8 (1.1)	—	41	10.8 (0.9)	--	62	18.1 (2.1)	--
*Hypertension > 140/90 mm Hg*^c^^d^												
With	87	8.1 (1.7)	0.56	144	10.3 (1.7)	0.69	177	13.2 (1.3)	0.67	189	22.0 (3.2)	0.045
Without	103	9.0 (0.7)	--	169	10.0 (0.9)	--	227	12.9 (0.6)	--	229	15.4 (1.4)	--

Age-standardized % are weighted. HbA1c = Hemoglobin A1c LDL = low density lipoprotein cholesterol

^*a*^Analyses among those with diabetes mellitus

^*b*^Analyses among those with diabetes mellitus or coronary heart disease

^*c*^Analyses among those with hypertension;

^d^indicated by systolic blood pressure > 140 or diastolic blood pressure > 90

### SNAP participation and emergency food use

Among participants income-eligible for SNAP, overall age-standardized self-reported participation was higher in participants with cardiometabolic conditions than those without (44.52% vs. 34.85%, p < .001). The percentage of income-eligible recipients who reported SNAP participation did not increase significantly over the study period (average APC in those with cardiometabolic conditions of interest +6.2%, 95% CI -4.5% to 18.1%). SNAP participation by time period and condition is shown in Table 3.

**Table 3:** Trends in SNAP participation among those income-eligible, by NHANES wave.

	2005–2006		2007–2008		2009–2010		2011–2012	
	Age-standardized % (SE) [N]	p	Age-standardized % (SE)	p	Age-standardized % (SE)	p	Age-standardized % (SE)	p
Diabetes Mellitus								
With	48.4 (4.1) [n = 182]	<0.001	41.7 (4.3) [n = 304]	0.52	51.1 (5.3) [n = 302]	0.11	49.6 (7.0) [n = 349]	0.45
Without	26.8 (2.7) [n = 915]	--	40.1 (3.1) [n = 1273]	--	44.5 (3.0) [n = 1524]	--	46.4 (3.1) [n = 1413]	--
Hypertension								
With	32.0 (4.2) [n = 425]	0.006	47.3 (3.7) [n = 655]	0.002	50.9 (3.1) [n = 702]	0.06	55.5 (2.8) [n = 705]	<0.001
Without	24.5 (2.8) [n = 672]	--	37.0 (3.7) [n = 922]	--	43.4 (3.5) [n = 1124]	--	40.3 (2.8) [n = 1057]	--
Coronary Heart Disease								
With	25.5 (8.1) [n = 109]	0.96	43.9 (5.7) [n = 140]	0.37	58.3 (7.1) [n = 155]	0.10	57.3 (6.6) [n = 145]	0.24
Without	27.8 (2.7) [n = 988]	--	40.3 (2.9) [n = 1437]	--	45.0 (2.9) [n = 1671]	--	47.1 (3.1) [n = 1617]	--
Congestive Heart Failure								
With	53.1 (8.9) [n = 46]	<0.001	n/a [n = 63]	n/a	76.3 (3.2) [n = 61]	<0.001	n/a [n = 84]	n/a
Without	28.0 (2.7) [n = 1051]	--	40.8 (2.9) [n = 1514]	--	45.3 (2.8) [n = 1765]	--	47.7 (3.0) [n = 1678]	--
Obesity								
With	36.2 (4.1) [n = 397]	0.001	46.2 (2.5) [n = 571]	0.038	49.6 (2.7) [n = 712]	0.017	50.8 (4.1) [n = 673]	0.19
Without	23.5 (2.3) [n = 669]	--	38.5 (3.4) [n = 970]	--	42.7 (3.4) [n = 1090]	--	45.9 (3.1) [n = 1057]	--
HbA1c > 9%^a^								
With	41.5 (5.3) [n = 30]	0.005	45.7 (4.5) [n = 46]	0.12	53.0 (5.7) [n = 49]	0.43	39.7 (8.9) [n = 67]	0.23
Without	28.2 (2.7) [n = 145]	--	41.1 (2.8) [n = 239]	--	45.3 (2.7) [n = 240]	--	47.2 (3.2) [n = 268]	--
LDL > 100 mg/dL^b^								
With	28.7 (4.4) [n = 70]	0.03	42.8 (3.2) [n = 85]	0.27	41.6 (3.4) [n = 103]	0.14	47.5 (5.3) [n = 113]	0.29
Without	39.1 (3.3) [n = 51]	--	39.3 (4.1) [n = 80]	--	45.5 (4.5) [n = 75]	--	44.8 (3.6) [n = 88]	--
Hypertension > 140/90 mm Hg^c^								
With	26.6 (6.1) [n = 219]	0.83	45.7 (6.9) [n = 315]	0.40	45.7 (4.3) [n = 311]	0.60	55.1 (5.7) [n = 320]	0.06
Without	28.9 (3.0) [n = 206]	--	41.3 (3.0) [n = 340]	--	46.1 (2.8) [n = 391]	--	45.4 (2.9) [n = 385]	--

Age-standardized % are weighted. HbA1c = Hemoglobin A1c LDL = low density lipoprotein cholesterol

N/a = unable to estimate given small sample size

^*a*^Analyses among those with diabetes mellitus

^*b*^Analyses among those with diabetes mellitus or coronary heart disease

^*c*^Analyses among those with hypertension

Overall, self-reported emergency food use was higher among those with cardiometabolic conditions (7.52% vs. 4.79%, p < .001), but did not increase significantly among those with a cardiometabolic condition during the study period (average APC 9.6% 95%CI -1.2% to 21.5%). Emergency food use by time period and condition is shown in S4 Table.

## Discussion

In this study of trends in food insecurity in a nationally representative sample of Americans from 2005 to 2012, we found that food insecurity increased throughout the study period, doubling from approximately 9% in 2005–2006 to 18% in 2011–2012. Food insecurity was significantly more common in those with, versus without, cardiometabolic conditions. However, those without cardiometabolic conditions still experienced a high prevalence of food insecurity, and the increase in food insecurity was similar for those with, versus without, the conditions of interest. We found no evidence of a downturn in food insecurity after economic recovery began in 2010—rather, food insecurity continued to rise. Compared with historical USDA data, the rates of food insecurity observed after 2009 are the highest recorded since measurement began in 1995.[16]

Because the foundation of therapy to prevent and manage the conditions studied is dietary modification[2–6], and because food insecurity incents dietary patterns that make cardiometabolic disease both more likely to occur and more likely to lead to complications[7], the growth of food insecurity has substantial public health implications. Among participants who did not have cardiometabolic conditions at the time of the study, the rise in food insecurity may be a risk factor for subsequent development of one or more of these conditions. High rates of food insecurity in diabetes and obesity, which pathophysiologically often precede coronary heart disease and congestive heart failure, may represent the ‘leading edge’ of further subsequent morbidity and mortality.

This study is consistent with and expands the results of prior work; several cross-sectional studies have demonstrated associations between food insecurity and diabetes, hypertension, and chronic kidney disease.[10, 11, 19, 20, 34–41] Prior studies of the relationship between obesity and food insecurity have yielded mixed results, with the strongest evidence of an association found in women[12]. Prior studies have not examined trends in food insecurity over time in clinical populations. In addition, the current study yields new evidence of an association between food insecurity and both coronary heart disease and congestive heart failure. Though the relationship between food insecurity and cardiometabolic conditions is likely bi-directional[7], ongoing food insecurity is detrimental to the management of cardiometabolic conditions regardless of the causal direction. Adults with cardiometabolic conditions and food insecurity face real financial barriers to dietary modification due to trade-offs between affording food, medications, and other basic needs[42, 43], even if food insecurity did not cause their condition. Therefore, the dramatic increase in food insecurity among those with cardiometabolic conditions is of particular importance for clinical management and public health.

There is growing interest in addressing social determinants of health through linkage of patients to community resources, as exemplified by the Center for Medicare & Medicaid Services’ recent Accountable Health Communities proposal.[25] Indeed, food insecurity is a major focus of that initiative. The estimates of SNAP and emergency food use reported here can help guide these linkage programs but have key limitations. NHANES estimates of SNAP participation are the best available for a nationally representative sample with data on health conditions, but they nevertheless underestimate participation rates provided by the USDA.[44] Reasons for this may include shame in reporting SNAP participation, which would tend to lower the ‘numerator’, and the fact that not all information necessary to calculate eligibility is available in the dataset, which would increase the ‘denominator’. If linkage of patients to community resources is to be widely pursued, dedicated studies should assess eligibility for, and participation in, available social programs. In the absence of such studies, the finding that self-reported SNAP participation is approximately five to fifteen percentage points higher in those with, versus without, the cardiometabolic conditions of interest, may be useful when combined with non-self-report participation data, in order to estimate current participation rates.

The observation that significant numbers of food insecure adults do not use nutrition support programs, such as SNAP and emergency food, reinforces the promise of linkage interventions. However, there will likely be challenges to implementing such interventions. While SNAP is effective in combating food insecurity[45], some of those with food insecurity may not be eligible, and the unstructured nature of the program combined with the relatively low value of the benefit may incent dietary strategies suboptimal for cardiometabolic disease management.[46] Food banks are a promising area of intervention[42], but are often overburdened, underfunded, and may have difficulty consistently sourcing the foods needed for cardiometabolic condition management. Moreover, food banks often work on a model of food distribution, providing a few days of food per month, that may not support the consistent changes in diet many Americans need. Collaboration between the healthcare system and the hunger safety net may help maximize the potential of this interventional approach.

The results of this study should be interpreted in the context of several limitations. Ascertainment of some clinical conditions of interest relied on self-report data. However, these interview items have been validated, and are commonly used to provide national disease prevalence estimates.[21–23, 47] Second, awareness of the importance of diet for their condition may cause participants with the conditions of interest to pay greater attention to food insecurity, increasing the observed differences in food insecurity between those with and without the conditions of interest. However, this would not alter the importance of recognizing and addressing food insecurity in the clinical management of these conditions. Finally, the small sample size of participants with some less common conditions (e.g. congestive heart failure), led to estimates with wide confidence intervals. These limitations are balanced by several strengths. The study made use of the high-quality epidemiologic surveillance data collected by NHANES, yielding estimates that are nationally representative. Additionally, because the methods of data collection were very similar throughout the study period, we can have greater confidence that the observed trends reflect true changes in food insecurity among non-institutionalized Americans.

## Conclusions

Food insecurity has reached historically unseen levels, doubling during the study period. It particularly affects those with cardiometabolic conditions, who most urgently need to follow a healthy diet. Because the appropriate prevention and management of diabetes, coronary heart disease, congestive heart failure, hypertension, and obesity all include dietary modification centered around increased consumption of fruits and vegetables and decreased consumption of sodium and highly processed foods, the increase in food insecurity has significant implications for the clinical care of these conditions, and the health of the public. There are opportunities to reduce food insecurity by using the hunger safety net, but there are also important challenges yet to be overcome. Making a concerted and expanded effort to address food insecurity may be a vital way to improve health in the United States.

## Supporting information

S1 Table Condition criteria. (DOCX)

S2 Table Food insecurity trends by gender, race/ethnicity, and education. (DOCX)

S3 Table Average annual percentage change (APC) in food insecurity prevalence, by condition, 2005–2012. (DOCX)

S4 Table Emergency food use trends by NHANES wave. (DOCX)
