# Supplementary material for: Trends in food insecurity for adults with cardiometabolic disease in the United States: 2005-2012
Source: PLoS One. 2017 Jun 7;12(6):e0179172. doi: 10.1371/journal.pone.0179172 (PMC5462405; doi:10.1371/journal.pone.0179172)
Supplement: S1 Table — (DOCX) [file pone.0179172.s001.docx]

S1 Table: Condition Criteria

**Diabetes**: Told by a doctor that you have diabetes, or plasma fasting glucose > 126 mg/dL, or Hemoglobin A1c > 6.5%, or use of glucose lowering medication other than metformin

**Hypertension**: Self-report of being told blood pressure is high on 2 or more doctor’s office visits, or self-report of taking a blood pressure lowering medication, or average of 3 systolic blood pressure readings > 140 mm/Hg, or average of 3 diastolic blood pressure readings > 90 mm/Hg

**Coronary heart disease**: Self-report of diagnosis of coronary heart disease or angina pectoris or a heart attack

**Congestive heart failure**: Self-report of congestive heart failure

**Obesity**: Body mass index > 30 kg/m^2^

**Uncontrolled hemoglobin A1c**: Among those with diabetes, Hemoglobin A1c > 9.0%

**Uncontrolled hypertension**: Among those with hypertension, average of 3 systolic blood pressure readings > 140 mm/Hg, or average of 3 diastolic blood pressure readings > 90 mm/Hg

**Uncontrolled low-density lipoprotein cholesterol**: Among those with diabetes or coronary heart disease, LDL cholesterol > 100 mg/dL, which was the goal-based treatment target used during the study dates
