# Supplementary material for: Trends in food insecurity for adults with cardiometabolic disease in the United States: 2005-2012
Source: PLoS One. 2017 Jun 7;12(6):e0179172. doi: 10.1371/journal.pone.0179172 (PMC5462405; doi:10.1371/journal.pone.0179172)
Supplement: S2 Table — (DOCX) [file pone.0179172.s002.docx]

| S2 Table: Food insecurity trends by gender, Race/ethnicity, and education | | | | | |
| --- | --- | --- | --- | --- | --- |
|  |  | NHANES Wave | | | |
|  |  | 2005-2006 | 2007-2008 | 2009-2010 | 2011-2012 |
|  |  | Age-standardized % (SE) | Age-standardized % (SE) | Age-standardized % (SE) | Age-standardized % (SE) |
| *Diabetes Mellitus* | | | | | |
|  | Women | 16.1 (5.0) | 16.2 (3.5) | 24.3 (6.1) | 30.3 (3.9) |
|  | Men | 14.1 (4.4) | 11.6 (3.0) | 17.7 (3.5) | 27.0 (4.1) |
|  | Non-Hispanic White | 9.0 (4.1) | 11.2 (3.2) | 15.5 (2.4) | 22.7 (7.2) |
|  | Non-Hispanic Black | 25.6 (5.0) | 11.4 (2.3) | 35.5 (7.5) | 28.3 (3.9) |
|  | Mexican-American and Other Hispanic | 15.9 (4.1) | 24.1 (3.8) | 30.2 (4.6) | 40.5 (7.0) |
|  | <HS diploma | 19.8 (5.9) | 24.6 (3.8) | 35.6 (8.4) | 39.4 (5.8) |
|  | ≥HS diploma | 13.9 (4.0) | 10.0 (2.3) | 17.1 (2.5) | 24.9 (3.2) |
| *Hypertension* | | | | | |
|  | Women | 9.4 (2.3) | 14.3 (1.5) | 21.8 (2.5) | 18.8 (2.4) |
|  | Men | 8.7 (0.9) | 10.3 (2.2) | 10.4 (1.5) | 20.4 (2.2) |
|  | Non-Hispanic White | 6.3 (1.6) | 9.6 (2.5) | 10.4 (1.5) | 15.0 (2.4) |
|  | Non-Hispanic Black | 19.2 (2.9) | 17.4 (2.2) | 26.7 (3.9) | 29.6 (2.4) |
|  | Mexican-American | 16.9 (5.5) | 22.6 (4.4) | 30.0 (3.7) | 28.3 (4.4) |
|  | <HS diploma | 20.9 (3.5) | 22.2 (3.0) | 29.4 (4.6) | 31.1 (3.7) |
|  | ≥HS diploma | 7.7 (1.3) | 9.7 (2.0) | 11.9 (1.4) | 16.9 (1.5) |
| *Coronary Heart Disease* | | | | | |
|  | Women | 7.3 (2.1) | 22.3 (4.6) | 31.1 (4.5) | 43.5 (8.8) |
|  | Men | 15.7 (5.5) | 19.0 (4.3) | 16.7 (7.8) | -- |
|  | Non-Hispanic White | 9.9 (5.3) | 15.6 (2.9) | 16.5 (8.0) | 31.3 (10.5) |
|  | Non-Hispanic Black | -- | 50.7 (5.5) | 29.1 (9.3) | 18.5 (5.5) |
|  | Mexican-American and Other Hispanic | 17.6 (7.7) | 21.7 (4.7) | 34.4 (5.1) | -- |
|  | <HS diploma | 7.8 (2.4) | 26.6 (7.1) | 34.1 (11.3) | -- |
|  | ≥HS diploma | 13.7 (4.3) | 18.2 (4.0) | 24.8 (2.0) | 27.7 (10.0) |
| *Congestive Heart Failure* | | | | | |
|  | Women | 16.3 (3.4) | -- | -- | -- |
|  | Men | -- | 12.3 (3.6) | -- | 28.8 (6.4) |
|  | Non-Hispanic White | -- | -- | -- | -- |
|  | Non-Hispanic Black | 22.9 (3.7) | -- | 19.8 (8.0) | 41.8 (4.6) |
|  | Mexican-American and Other Hispanic | -- | -- | -- | -- |
|  | <HS diploma | -- | -- | -- | 43.4 (4.8) |
|  | ≥HS diploma | 15.5 (3.4) | 13.0 (4.8) | 6.2 (2.3) | -- |
| *Obesity* | | | | | |
|  | Women | 11.6 (1.6) | 14.2 (1.6) | 19.8 (1.7) | 22.4 (1.9) |
|  | Men | 6.7 (0.8) | 9.6 (1.8) | 11.1 (1.4) | 18.2 (1.7) |
|  | Non-Hispanic White | 5.6 (1.2) | 8.5 (1.6) | 9.6 (1.2) | 16.1 (1.9) |
|  | Non-Hispanic Black | 16.6 (2.2) | 15.1 (1.7) | 25.5 (2.8) | 26.4 (2.9) |
|  | Mexican-American | 20.8 (3.3) | 22.9 (3.1) | 31.0 (2.5) | 29.8 (3.8) |
|  | <HS diploma | 17.9 (2.8) | 22.6 (2.7) | 33.5 (3.4) | 33.9 (1.8) |
|  | ≥HS diploma | 7.6 (1.1) | 8.9 (1.1) | 11.1 (1.0) | 17.4 (1.6) |
| *HbA1c > 9%* | | | | | |
|  | Women | 33.3 (4.8) | 25.1 (8.5) | 34.1 (3.7) | 32.7 (6.1) |
|  | Men | 28.5 (6.9) | 32.8 (4.6) | 38.8 (3.7) | 16.2 (5.2) |
|  | Non-Hispanic White | 31.0 (8.0) | -- | -- | 29.9 (6.9) |
|  | Non-Hispanic Black | 41.3 (7.8) | -- | 53.7 (9.5) | 19.4 (5.3) |
|  | Mexican-American and Other Hispanic | 19.0 (4.5) | 23.7 (9.8) | 45.3 (3.3) | -- |
|  | <HS diploma | 31.8 (10.0) | 48.2 (8.8) | 36.0 (5.2) | 28.9 (5.3) |
|  | ≥HS diploma | 31.9 (2.5) | . (.) | 38.5 (4.9) | 23.8 (5.3) |
| *LDL > 100 mg/dL* | | | | | |
|  | Women | 21.6 (9.3) | 12.7 (2.3) | 32.0 (6.1) | 28.2 (6.3) |
|  | Men | 22.3 (3.8) | 13.7 (4.9) | -- | 26.9 (5.8) |
|  | Non-Hispanic White | 17.8 (9.9) | 11.8 (4.8) | 36.0 (3.4) | -- |
|  | Non-Hispanic Black | -- | 10.4 (3.2) | -- | 14.6 (3.1) |
|  | Mexican-American and Other Hispanic | 36.4 (9.4) | 25.0 (5.0) | 25.2 (7.9) | 45.7 (7.4) |
|  | <HS diploma | 21.8 (6.5) | 24.6 (5.0) | 47.2 (6.8) | -- |
|  | ≥HS diploma | 23.3 (9.1) | 7.8 (1.8) | 19.0 (7.1) | 29.4 (4.2) |
| *Hypertension > 140/90mm/Hg* | | | | | |
|  | Women | -- | 12.0 (2.5) | 21.0 (5.8) | 22.1 (3.8) |
|  | Men | 8.7 (1.7) | 9.0 (1.3) | 10.7 (1.4) | 20.3 (4.2) |
|  | Non-Hispanic White | 5.8 (2.3) | 8.3 (2.2) | 7.4 (1.8) | 12.9 (3.7) |
|  | Non-Hispanic Black | 16.3 (3.7) | 15.3 (2.7) | 25.6 (6.8) | 34.6 (3.4) |
|  | Mexican-American and Other Hispanic | 12.2 (4.2) | 13.9 (2.0) | 26.4 (4.5) | 38.5 (8.4) |
|  | <HS diploma | 18.0 (4.3) | 13.3 (2.0) | 26.4 (3.5) | 34.1 (7.9) |
|  | ≥HS diploma | 5.9 (1.5) | 8.7 (2.2) | 9.0 (1.3) | 19.2 (3.3) |
| Age-standardized % are weighted. HbA1c = Hemoglobin A1c. Analyses among those with Diabetes Mellitus. LDL = low density lipoprotein, analyses among those with diabetes mellitus or coronary heart disease. -- = unable to calculate given sample size | | | | | |
