# Supplementary material for: Trends in food insecurity for adults with cardiometabolic disease in the United States: 2005-2012
Source: PLoS One. 2017 Jun 7;12(6):e0179172. doi: 10.1371/journal.pone.0179172 (PMC5462405; doi:10.1371/journal.pone.0179172)
Supplement: S3 Table — (DOCX) [file pone.0179172.s003.docx]

| S3 Table: Average Annual Percentage Change (APC) in Food Insecurity Prevalence, by Condition, 2005-2012 | | |
| --- | --- | --- |
|  | Average APC | 95% CI |
| *Diabetes Mellitus* | | |
| With | 14.1 | (-0.9 to 31.4) |
| Without | 10.1 | (6.3 to 14.1) |
| *Hypertension* | | |
| With | 13.5 | (11.8 to 15.3) |
| Without | 8.6 | (1.9 to 15.8) |
| *Coronary Heart Disease* | | |
| With | 12.8 | (-3.2 to 31.3) |
| Without | 10.6 | (5.6 to 15.7) |
| *Congestive Heart Failure* | | |
| With | 10.6 | (-17.4 to 48.1) |
| Without | 10.7 | (7.0 to 14.6) |
| *Obesity* | | |
| With | 14.0 | (12.6 to 15.5) |
| Without | 8.5 | (3.9 to 13.2) |
| *HbA1c > 9%* | | |
| With | -0.4 | (-23.2 to 29.3) |
| Without | 10.6 | (7.4 to 13.9 ) |
| *LDL > 100 mg/dL* | | |
| With | 13.7 | (-5.2 to 36.3) |
| Without | 9.7 | (-8.7 to 31.9) |
| *Hypertension > 140/90 mmHg* | | |
| With | 18.0 | (3.2 to 35.1) |
| Without | 9.8 | (5.4 to 14.3) |
| HbA1c = Hemoglobin A1c. Analyses among those with Diabetes Mellitus. LDL = low density lipoprotein, analyses among those with diabetes mellitus or coronary heart disease. | | |
