# Supplementary material for: Trends in food insecurity for adults with cardiometabolic disease in the United States: 2005-2012
Source: PLoS One. 2017 Jun 7;12(6):e0179172. doi: 10.1371/journal.pone.0179172 (PMC5462405; doi:10.1371/journal.pone.0179172)
Supplement: S4 Table — (DOCX) [file pone.0179172.s004.docx]

| S4 Table: Emergency food use trends by NHANES wave | | | | | | | | |
| --- | --- | --- | --- | --- | --- | --- | --- | --- |
|  | 2005-2006 | | 2007-2008 | | 2009-2010 | | 2011-2012 | |
|  | Age-standardized % (SE) | p | Age-standardized % (SE) | p | Age-standardized % (SE) | p | Age-standardized % (SE) | p |
| *Diabetes Mellitus* | | | | | | | | |
| With | 9.2 (2.8) | 0.11 | 8.1 (1.5) | 0.03 | 13.2 (1.8) | 0.005 | 12.6 (2.0) | 0.01 |
| Without | 4.8 (0.6) | -- | 5.0 (0.6) | -- | 6.8 (1.0) | -- | 7.8 (1.1) | -- |
| *Hypertension* | | | | | | | | |
| With | 6.8 (1.0) | 0.002 | 6.9 (1.0) | 0.001 | 9.6 (1.0) | <0.001 | 12.4 (1.9) | 0.002 |
| Without | 4.2 (0.6) | -- | 4.1 (0.5) | -- | 6.5 (1.0) | -- | 7.0 (1.1) | -- |
| *Coronary Heart Disease* | | | | | | | | |
| With | 17.0 (5.9) | 0.03 | 18.3 (5.8) | 0.02 | 14.7 (3.2) | 0.008 | 11.8 (3.8) | 0.32 |
| Without | 4.7 (0.6) | -- | 5.0 (0.6) | -- | 7.1 (0.9) | -- | 8.1 (1.1) | -- |
| *Congestive Heart Failure* | | | | | | | | |
| With | 22.0 (8.2) | 0.03 | 17.1 (4.5) | 0.009 | 4.9 (2.9) | 0.50 | n/a | n/a |
| Without | 4.8 (0.6) | -- | 5.1 (0.6) | -- | 7.3 (0.9) | -- | 8.1 (1.1) | -- |
| *Obesity* | | | | | | | | |
| With | 7.3 (1.0) | <0.001 | 6.9 (1.0) | 0.004 | 9.0 (0.9) | <0.001 | 10.6 (1.3) | <0.001 |
| Without | 3.8 (0.6) | -- | 4.5 (0.5) | -- | 6.3 (0.9) | -- | 6.9 (1.2) | -- |
| *HbA1c > 9%* | | | | | | | | |
| With | 20.2 (5.4) | 0.009 | 8.4 (2.6) | 0.12 | 24.7 (5.6) | 0.003 | 7.3 (2.4) | 0.88 |
| Without | 5.0 (0.6) | -- | 5.1 (0.5) | -- | 7.3 (0.9) | -- | 8.0 (1.1) | -- |
| *LDL > 100 mg/dL* | | | | | | | | |
| With | 6.0 (1.1) | 0.89 | 5.0 (1.0) | 0.40 | 7.1 (1.3) | 0.39 | 8.9 (1.4) | 0.87 |
| Without | 6.1 (1.4) | -- | 6.1 (0.9) | -- | 5.9 (0.9) | -- | 9.5 (2.0) | -- |
| *Hypertension > 140/90 mm/Hg* | | | | | | | | |
| With | 6.2 (1.3) | 0.31 | 4.8 (1.1) | 0.79 | 8.6 (1.2) | 0.37 | 10.6 (2.2) | 0.14 |
| Without | 4.8 (0.6) | -- | 5.1 (0.6) | -- | 7.1 (0.9) | -- | 7.9 (1.1) | -- |
| Age-standardized % are weighted. HbA1c = Hemoglobin A1c LDL = low density lipoprotein cholesterol  N/a = unable to estimate given small sample size  *^a^*Analyses among those with diabetes mellitus  *^b^*Analyses among those with diabetes mellitus or coronary heart disease  *^c^*Analyses among those with hypertension | | | | | | | | |
